# Using co-design to understand consumer’s health information-seeking behaviours and design preferences for a new digital clinical dashboard in aged care

**DOI:** 10.1186/s12877-024-05581-2

**Published:** 2024-12-04

**Authors:** Amy D Nguyen, Laura Dodds, Kristiana Ludlow, Melissa Baysari, Rosa Comi, Wu Yi Zheng, Johanna I Westbrook

**Affiliations:** 1 Centre for Health Systems and Safety Research, Australian Institute of Health Innovation, Macquarie University, Sydney, NSW 2109 Australia; 2 St Vincent’s Clinical Campus, UNSW Sydney, Sydney, NSW Australia; 3 Centre for Health Services Research, University of Queensland, Brisbane, QLD Australia; 4 Biomedical Informatics and Digital Health, Faculty of Medicine and Health, The University of Sydney, Sydney, Australia; 5 Faculty of Medicine, Health and Human Sciences, Macquarie University, Sydney, NSW Australia; 6 Black Dog Institute, UNSW Sydney, Sydney, NSW Australia

**Keywords:** Digital health, Aged care, Health information technology, Information seeking, Stakeholder participation, Dashboard, Co-design

## Abstract

**Background:**

Little is known about the information needs of older people and their family caregivers, particularly around medication management. This is largely because this population are infrequently consulted in research. Health technologies such as digital dashboards can present comprehensive and timely data summaries to improve knowledge and guide decision-making.

**Objectives:**

The aim of this study was to understand current information seeking-behaviours of aged care service users and their families, and their preferences for the presentation of these types of information to support the co-design of a digital aged care dashboard.

**Methods:**

Aged care clients (community and residential aged care) and their family members were invited to participate in semi-structured, one-on-one interviews conducted over the telephone. The interviews explored how participants sought health information, how this information supported their clinical decision-making, and how they would want this information to be presented via a digital dashboard. Interviews were audio-recorded, transcribed verbatim and independently analysed using an inductive content approach by three reviewers to identify prominent categories.

**Results:**

Aged care clients and family members sought health information from healthcare providers, aged care facilities and the Internet. Information regarding medications, medical conditions and an overview for family members were of particular importance to participants. Participants saw the usefulness of a digital dashboard displaying relevant information, and provided suggestions regarding presentation of this information. This included the use of large text, representative colours, symbols and graphs. Participants recommended being able to tailor the dashboard to individual end-users.

**Conclusions:**

By engaging aged care clients and their families in research, it was found that they had positive views about a digital dashboard that presented clinical information and was readily accessible. Such a dashboard could complement their current practices of information-seeking. However, for the dashboard to be effective for this population, several suggestions for its design were raised. Understanding the information-seeking behaviours of aged care clients and their families, together with knowledge of the information that is important to them, and then subsequent incorporation of this information into a digital dashboard that reflects their design preferences, could lead to more informed decision-making in this population.

**Supplementary Information:**

The online version contains supplementary material available at 10.1186/s12877-024-05581-2.

## Introduction

In the aged care sector, access to information, and therefore being informed, has been shown to support better decision-making regarding an aged care service-users’ care [1]. Difficulties in accessing clinical information about those receiving aged care services is evident, and it is unclear what types of information are important to aged care clients and their families [1–4]. Digital dashboards have been used to combine multiple data sources for predictive analytics in order to present more useful information, and are used across a variety of healthcare settings to improve patient care [5]. They allow for dynamic summaries of an individual’s information (e.g. medications, medical history, biometrics) and can be used to assist decision-making using text and visual features (e.g. alerts to deconditioning, high risk of injury or harm). In aged care, dashboards are relatively new, with some being developed for home care [6–13], long-term care [14–19] and respite services [15, 17], with the majority facilitating client assessments and service planning (e.g., initial assessments, co-ordinating care transitions) [20, 21]. Guidelines for dashboard development specify that dashboards should be customisable and actionable, have high-level information aggregation, adhere to conventions, have a minimalist design, include built-in guidance and training, and result in workload reduction [22]. However, little is known about how to translate these guidelines into practice. Evaluation of dashboard usability and acceptability in aged care is common post-implementation or after a specified trial period [21]. This approach is ‘too late’ as it limits the versatility of the design of the dashboard to be used in real-world situations and integrated into routine work processes [23, 24].

With the introduction of dashboards within aged care settings, acceptability and usability issues have emerged [21]. Heterogeneity of end-users, functional and hardware problems, and the need for training tailored to different computer literacy levels and learning preferences, are common barriers to dashboard adoption within broader healthcare contexts [7, 21, 23, 25]. Within aged care specifically, there are a variety of stakeholders and healthcare services (hospitals, medical specialists, allied health professionals, dental and pharmaceutical services, and palliative care) involved in addressing escalating patient care needs over a long period of time [26]. Therefore, dashboard customisability and balancing multiple end-user preferences and dashboard objectives (e.g., clinical decision-making or interactive health technology for patients [27, 28]), can be a substantially greater challenge in aged care. Notably, working conditions and staffing challenges (e.g., understaffed, overworked, and undertrained workforce [29]), as well as the traditionally lower levels of information technology uptake in the aged care sector [30], do not support streamlined integration of digital dashboards and may be additional barriers to dashboard adoption in aged care.

Participatory approaches like co-design may be a potential solution to ensure there are significant benefits of developed tools for stakeholders. A co-design research approach involves the meaningful engagement of end-users throughout all stages of research [31] and has been widely used when developing digital technologies for healthcare settings [32]. The adoption of co-design principles in aged care generally has been minimal [33]. However, co-design interventions involving older adults have been shown to lead to new information, significant and beneficial adjustments in design, and a sense of participation [34–37]. Effective adoption of co-design can minimise the challenges associated with balancing stakeholder needs, can facilitate relevant and translatable outcomes for end-users, positively influence clinical outcomes in patient safety, and improve the function of health systems [31]. Applying co-design techniques to dashboard design could ensure that visualisations, content, and functionality complement current workflow processes and strengthen uptake, assist with ease and efficiency of task completion, and support decision making and immediate action for end-users [7, 38].

This current study formed part of a larger research project that aimed to co-design, implement and evaluate an aged care digital dashboard comprising: (a) integrated client data, (b) predictive risk models in real-time, and (c) decision support to reduce identified risks. A protocol for this research project can be found here: https://bmjopen.bmj.com/content/11/8/e048657 [39]. In this article, we use the term “client” to collectively refer to residents accessing residential aged care services and older adults accessing community/home care services. The co-design process of this project involve (i) direct observations of work routines of aged care staff; (ii) interviews and focus groups with aged care staff, clients and family members, and general practitioners (GPs) to explore how health information is currently sourced and used, as well as visual and functional design features of the dashboard; (iii) iterative co-design groups or workshops with aged care staff, GPs, aged care clients, and informal caregivers to design and refine the decision support features for the risk indicators. This study reports on one of the initial dashboard co-design phases, specifically interviews with aged care clients and family members. The study aimed to explore aged care clients’ and family members’ preferences and ideas to inform the design and development of a new digital dashboard. This study was guided by two research questions:

1. What are aged care clients’ and family members’ current processes of seeking and identifying health information?
2. What are aged care clients’ and family members’ preferences and ideas for the content, appearance and delivery of the new dashboard?

## Methods

### Participants and setting

The research team partnered with a large aged care provider in NSW, Australia. The aged care provider offers community care (home care) and residential aged care services to Greater Sydney, Central Coast, Illawarra Shoalhaven, Hunter and Northern Inland regions. The participant eligibility criteria were: (1) clients currently receiving community aged care or residential aged care services from the partner aged care provider, or; (2) family members of clients receiving this care; (3) the ability to communicate in, and understand, plain English; and (4) the capacity to provide informed consent.

### Recruitment

The research team worked with aged care managers and staff members (community and residential aged care) to facilitate identification of eligible participants via purposive sampling methods. Managers and staff members provided study invitation letters and participant information and consent forms to potential participants. Aged care clients and family members who expressed interest in the study were contacted by the research team by phone to obtain consent and schedule an interview. Participants did not receive any form of remuneration. Recruitment remained open for 14 months. In this period, individual interviews were completed with aged care clients and their family members.

### Data collection

Participants engaged in one-on-one interviews covering the following topics: information-seeking, decision-making, and dashboard preferences. Prior to the interviews, the purpose of the study was explained both in written format and verbally to participants, and a combination of written and verbal consent was obtained. During the interviews, participants were asked about their information-seeking behaviours such as what types of information they sought and where they found them, and how this information supported decision-making. A description of what a digital dashboard is, including who they can be used by and what clinical outcomes dashboards can support, e.g., identification of at-risk patients, was provided. Participants were then asked about their preferences for a dashboard in terms of content, functionality and presentation, in the context of a new aged care dashboard for consumer use. Interviews were conducted over the telephone, by one of three researchers (ADN, LD, KL), audio-recorded, professionally transcribed verbatim and de-identified to ensure confidentiality.

### Analysis

Transcripts were analysed using inductive content analysis [40]. Each transcript was analysed by at least two researchers (ADN, LD, KL). Transcripts were read for familiarity and open codes were independently applied by each researcher using NVivo (V12). Each researcher looked for patterns in the data and major and minor categories were identified by grouping codes with similar semantics. Reviewers then convened to discuss emerging major categories and develop an analysis codebook for each setting. The transcripts were then assigned to LD who coded all transcripts using the codebook.

## Results

Study participants comprised aged care clients (aged care residents and those receiving home and/or community care services) (*n* = 22) and their family members (*n* = 4), with 20 from residential aged care settings and 6 from community services overall. Four main categories were identified across the participant sample, guided by the interview topics: (1) Information-seeking; (2) Information accessibility; (3) Decision-making; and (4) Dashboard preferences (Table 1). For two of the main categories, “information-seeking” and “dashboard” preferences, minor categories were identified.

**Table 1:** Categories identified from participant responses

Main Category	Information-seeking	Information accessibility	Decision-making	Dashboard preferences
*Minor categories*	Information sources used			Dashboard content
	Reliability and accuracy of information			Dashboard appearance
	Volume of information			Dashboard delivery

### Information-seeking

#### Information sources used

For community clients, common sources of health information were reported to be GPs and pharmacists. Participants often spoke about the established and trusting relationship they had with these healthcare professionals.

*“We’ve seen the same GP for 30 years plus and he is absolutely brilliant*,* I mean in terms of his diagnostic skills… I think he’s been an absolute lifesaver… I can’t speak highly enough in terms of the healthcare that our GP has given us and honestly*,* I’m not exaggerating*,* I think*,* if it weren’t for him*,* we wouldn’t be here*,* either of us.”* (Community client 8).

*“I’ve known him [pharmacist] for 30 odd years. I just accept what he says*,* but if I’ve got a question*,* I will ask him*,* and he tells me immediately. We’ve got a very good rapport.”* (Community client 6).

Other sources of information described by community clients included written materials (e.g., brochures, printouts from GPs), family and friends, nurses, consumers groups, and support groups. The most common system reported by community clients for collating health information, such as medicines, was a static, printed list provided by their GPs. Clients explained that any changes to their medications required a new list to be printed, or handwritten changes to be made to their current lists.

*“I did have a list from the doctor. Every time we added a new medication*,* I’d ask the doctor to add it to the list and then give me a copy of his list of medications*,* because when he [husband] went to hospital … they wanted a list of those as well … I found that very useful. I always had a copy because I couldn’t remember the names of things. They had very long names.”* (Community client 19).

Another common system used was a Webster pack (pre-packed medication system that organises several days of medications into “blister” compartments to be taken at various times of the day, usually prepared by a pharmacist) or a medicines container that community clients or their families organised themselves.

For aged care residents and family members, the staff members and formal carers within aged care facilities were the most common source of information.

*“The nurse will sit down and explain it to me*,* ‘This is what’s wrong and this is what’s going to happen*,*’ and they’ll sit there and then explain it to me fully*,* so I do understand it.”* (Residential family member 2).

Among community clients, aged care residents and family members, the Internet was mentioned as a common source of general information, including searching for medical symptoms, treatments, and medication side effects.

*“If I saw a new medication and I wanted to know what it was*,* I would simply look it up online on MIMS or something like that.”* (Residential family 1).

### Reliability and accuracy of information

Unlike community care clients, aged care residents did not mention a system for tracking events or changes in medications. Instead, family members expressed concerns about the collation and storage of consistent health information in residential aged care. While family members were aware of the My Health Record (a national online repository of health information), they were unsure what information it contained.

*“I’ve never actually seen my mum’s file at the* [aged care facility], *but the trouble is that over the last few years she’s been to a number of medical centres … So*,* there’s been a few transfers between GPs*,* and I’m not convinced that all of the information has been transferred and I have no idea how I can check that*,* whether they [residential aged care facility] actually have the full amount of information*,* and that’s something that really concerns me.”* (Residential family member 1).

Family members said they often relied on the aged care facility for information about their relative, however, some were doubtful of the accuracy and timeliness of certain information that was conveyed to them.

*“There’s a bit of inconsistency about what they’re meant to tell me and what they do tell me*,* and sometimes I’m not even sure what information I’m meant to get.”* (Residential family 1).

This concern extended to documented information within facility records. Family members and aged care residents lacked confidence in the level of accurate and timely communication between external health professionals involved in the resident’s care (e.g., GPs, specialists) and the aged care facility.

*“The only problem is that if there is a need for some medication to be delivered promptly*,* and actioned promptly*,* sometimes it’s done*,* say a doctor visits on Friday and Tuesday*,* there is a delay on Saturday*,* Sunday and Monday before the doctor comes in to sign off the chart.”* (Aged care resident 1).

### Volume of information

When asked about the amount of information participants preferred to receive about their care and health, opinions varied. Community clients wanted a great amount of detail; some stating it was “their body” and therefore, they wanted to be as informed as possible. Whereas aged care residents only wanted information if they requested to be informed about their condition, otherwise they described preferring to avoid information overload unless the clinician deemed it necessary.

*“I’m not one to be told something*,* go away and just say ‘Yes doctor’. I always inquire*,* make sure I know everything I can possibly find out about a disease and how it’s treated*,* and whether the treatment I’m being told about is suitable for me*,* whether I want to go ahead with it. I like to thoroughly investigate things.”* (Community client 19).

*“I guess there are some things they [doctor] think*,* well*,* she wouldn’t want all this information*,* it would be a bit much*,* but I guess they do it according to how the patient seems.”* (Aged care resident 1).

Some community clients emphasised that they wanted information to be detailed, but provided in simple language. Other community clients, and most aged care residents, wanted simple summaries that highlighted the most important information they needed to know.

*“I like to listen to what they’re [GP] saying*,* I’d like to ensure they are answering the question. And I don’t need to know the full nitty-gritty of it*,* just the basic information.”* (Community client 26).

### Information accessibility

Most community clients and aged care residents expressed that they were able to access health and care information when required.

*“If I want to see my medication chart*,* I just ask for it*,* ask the carer for it*,* they go to the RN and they bring it to me. There are one or two drugs that are on PRN [pro re nata*, i.e.,* as needed]*,* and as and when I need these*,* these are usually delivered extremely promptly.”* (Aged care resident 2).

Some community clients noted that while staff members were extremely helpful, clients had experienced delays in getting in contact with staff.

*“The lady who is allocated to me… She doesn’t return the calls but there’s somebody who – maybe her boss. I think this girl [staff member] might be overladen and overloaded with clients*,* but when she doesn’t ring me back*,* this other girl rings me back and she’s more than helpful*,* is very knowledgeable and saves the day.”* (Community client 26).

Information was not easily sourced by family members, particularly in relation to medications. For example, one family member made sure to check their billing statements every month to see what was being ordered from the pharmacist, whilst for others, it was difficult to access this information all together.

*“I’m not sure exactly what the medication is for my partner. I know she takes certain different things every day – eight different things every day*,* but I’m not sure exactly what they are. I’d like to check on that*,* if I had a resource I could do it I’d be doing it. But*,* I mean*,* if it was on a spreadsheet*,* I could see*,* yes*,* this tablet does this for the person*,* this tablet does this for the person; that would be a lot better for me.”* (Residential family 2).

Family members expressed that at times, the knowledge they considered important was withheld, stating that the information they received was often trivial or only focused on negative incidents.

*“What I find is that often they communicate really well on trivial things but when it comes to things that are really quite important suddenly*,* I don’t get any information.”* (Residential family 1).

*“We hear about if something is going wrong*,* but we don’t get much – we don’t hear so much on the positive side*,* what’s actually happening or what they’re doing or what they’re able to achieve and I think I’d like something – I think I would like to hear a bit more often about that.”* (Residential family 3).

### Decision-making

Most community clients wanted to be involved in making decisions about their care, often in collaboration with their family members and healthcare providers.

*“Mostly I leave it up the GP. Occasionally I might question things*,* or see if there’s any alternative*,* or something better*,* or whether medication ought to be reduced and the implications of it. So that’s a full and open discussion.”* (Community client 13).

Although aged care residents also made their own decisions about their care, family members were more heavily involved and consulted in the decision-making process, often taking an advisory role to the resident.

*“I am quite involved. [Resident] prefers me to know what’s happening*,* even though… when you speak to her*,* you’ll find she’s mentally quite alert and she’s really doing incredibly well for 97*,* and she can make a lot of decisions herself*,* but she often gets confused by things.”* (Residential family 1).

### Dashboard preferences

Participants had varying comfort levels with using technology, with some having limited experience with technology, and one with experience in the information technology sector. What a clinical dashboard is and what it can entail and achieve was explained to participants. They were then asked about their suggestions for the presentation and functionality of a new aged care digital dashboard. Participants shared their preferences and ideas regarding the content, appearance, and delivery of the dashboard.

### Dashboard content

Regarding dashboard content, participants discussed the types of health and aged care information they wanted included in the dashboard. Access to medication-related information was a key priority for participants. Clients living in the community shared the view that incorporating information about the type, dose, and potential side effects of medications, as well as reminders to take their medication (especially when it has changed from their current methods) in the dashboard would be valuable.

*“Medications*,* that would probably be a good one. Sometimes I forget to take my heart medication in the afternoon so*,* yes*,* that would be a good one.* (Community client 8)

Community clients also requested pathology results, general health information (e.g., information about the COVID-19 pandemic), personal medical appointments, doctors’ notes, end-of life care, non-pharmacological treatments (e.g., physiotherapy) and available aged care provider services, to be included in the dashboard. One client suggested having a banner listing the types of services offered by the aged care provider.

*“Some things you might not know about [aged care provider]*,* what they actually do offer*,* and maybe just have some at the bottom*,* or somewhere along the top of the homepage and just list the sorts of things that you can get through [aged care provider]*,* like home care.”* (Community client 19).

Specific to residential aged care, residents and their family members were interested in incorporating information into the dashboard that provided a holistic view of the resident. For example, general health updates (e.g., incidents, aids, wounds) and day-to-day functioning (e.g., upcoming events, food options, eating and sleeping habits, and participation in lifestyle activities within the aged care facility) were suggested.

*“To remind me of key events*,* like for example … we have the hydrotherapy on Monday morning. Tuesdays and Wednesdays are normally free. I have bible study on Thursday afternoon. And support group meeting every second week probably now*,* on a Friday … So that’s kind of*,* typical example of my needs.”* (Aged care resident 2).

*“Maybe what sort of things*,* activities [resident] might be involving herself in and food*,* yeah. And they’re things that I ask her but — like*,* for somebody who’s just logging in to look at it*,* I think that something like that*,* with that information gives a pretty good picture. Yeah*,* and health issues.”* (Residential family 4).

This overarching view or summary was deemed especially important for family members of aged care residents with cognitive impairment or dementia.

*“That’s something I’d like a bit more clarity on exactly what [resident] does … I’m not sure about that because he often says*,* “I’ll go for a little walk after lunch”… but because he’s got dementia*,* I don’t know whether he actually does that*,* when it comes around to it or whether that’s all in his mind.”* (Residential family 3).

Family members also viewed the dashboard as a potential method of communication between those involved in the care of the resident and a way to review the accuracy of information.

*“I guess because I saw this as being mainly about getting sort of health information and communication of health information about my mum*,* so I guess I was mainly looking at how accurate communication is between residents*,* the medical professionals*,* the facility and residents’ relatives.”* (Residential family 1).

While some community clients wanted to have contact numbers to talk with a nurse or other professional about their dashboard, most said they would prefer to talk about this with their personal GP.

Most participants in both community and residential care requested the option of obtaining more detailed information about their specific health condition(s), medications, or change in health status. They proposed the use of hyperlinks or a way to click through to more details.

*“I think more summarised*,* and perhaps … if there was something that we wanted a bit more info on*,* could we click on something to go further. But sort of immediately summary*,* but is there some way of go into detail if we felt we needed it?”* (Residential family 3).

Aged care clients and their family members were also asked about how frequently information within the dashboard should be updated. Residents and family members suggested that there should be updates when something changes, particularly in relation to medications, so that these modifications can be actioned promptly. Similarly, community clients also wanted information updated when they had a change in their treatment.

*“I think to some extent I would find it useful to have it updated regularly with whatever the doctor has prescribed or changed in the way of prescriptions or – so that I know what’s happening medically and to see it used for communication*,* not just with me but between other health professionals.”* (Residential family 1).

### Dashboard appearance

When asked about information presentation on the dashboard, a common response from both community clients and aged care residents was for text to be simple and jargon free. All participants preferred information to be summarised with minimal text used on the dashboard, to avoid clutter.

*“I think*,* you want to present the facts*,* but … you wouldn’t need too much medical jargon. You need it to be in terms that people understand but*,* you know*,* sometimes you can get too much information.”* (Community client 10).

Participants spoke about the need for the text to be easy to read. There was a consensus that a standard 12-point font was acceptable, with some community clients suggesting that the American Blind Association font is used, or that the designers consult with societies such as the Blind Society when making decisions about text presentation. Other suggestions about the dashboard appearance included avoiding coloured backgrounds and glary or faint text.

*“I like a darker print*,* and not so bright a background*,* because I find the glare affects my eyes*,* but so long as the text is just a little bit larger and it’s clear*,* not too faint.”* (Community client 19).

Community clients, aged care residents and their family members were positive about the use of colours, particularly to highlight important information. Participants often referred to the ‘traffic light system’, where red indicates the most urgent tasks or when information was outside of thresholds (e.g., test results), amber represents less urgent tasks, and green signals that information does not require attention.

*“If red came up*,* oh*,* there’s something to worry about. If it was green or blue*,* I would think*,* oh*,* well that’s something that’s good going on.”* (Community client 8).

Most participants reported a preference for the use of graphs to represent test results, such as blood glucose levels over time. However, the use of symbols and pictures triggered both positive and negative responses. Participants emphasised that visual features (colours, symbols, images and graphs) needed to be used appropriately and sparingly.

*“Oh*,* not stupid symbols*,* like smiling faces and things. No*,* don’t give me anymore of that*,* please. Sensible*,* relevant symbols.”* (Aged care resident 1).

Community clients, aged care residents and their family members also suggested that graphs, symbols and alerts be accompanied by some explanatory text to clarify the information presented. One family member mentioned this would be particularly useful for new staff who are unfamiliar with a resident’s health status.

### Dashboard delivery

When asked how they would access and use the dashboard, participants reported a preference for accessing the dashboard via an online platform. One community client explained why a digital version of the dashboard would be beneficial:

*“I just remembered when I had to go unexpectedly to hospital*,* and they wanted a list of my medications and I happened to have it written down in my wallet. But you wouldn’t need that if it’s all electronic*,* would you?”* (Community client 10).

Aged care residents and family members shared a similar view, and explained they would access the dashboard on multiple devices for example, a desktop computer, laptop or iPad and via different modalities such as via email, website or mobile application.

*“I think if it was a link to a portal somewhere*,* where I could do that. That would be helpful.”* (Aged care resident 2).

*“I think an app*,* because when we get links if they get too far back in the message stream*,* we can’t find them… So*,* an app would be – for me at my level of understanding*,* would be an easier thing to use.”* (Residential family 3).

A number of community clients and aged care residents raised concerns about the feasibility of a digital dashboard. Some community clients said they would want a printout for easier, non-technology-based presentation of the dashboard information, and others suggested offering clients the option of either paper-based or electronic delivery. Aged care residents suggested a digital dashboard would be more useful for their family members than for themselves. Residential aged care clients and family members proposed that one-on-one training or dashboard viewing with family members present may be beneficial to enhance the feasibility of the dashboard for this user group.

## Discussion

This study provides novel insights into how aged care consumers access information and how they would like this information presented to them. Additionally, the preferences and design ideas of older adults receiving aged care services and their family members for a new digital dashboard to enhance the quality of their care were revealed. Interviews showed that older adults receiving aged care services and their family members were able to foresee benefits of implementation of a digital clinical dashboard in practice.

This study uncovered some key information needs for aged care residents and their family members. In particular, residents and families do not completely trust the health information they are currently given, largely due to the information being seen as out-of-date by the time it reached family members. Additionally, family members stated that they were unable to access information that was important to them, particularly around medications. Similar results have been reported by others [1–3], with a Swedish study showing that older people receiving aged care services in the community feel anxious and insecure when they do not receive enough information, such as when they are not provided the opportunity to contact their healthcare providers, do not have the tools that allow them to find medication information or have consultations that are too-short [4]. It has been suggested that residents and family members receiving poor information can be attributed to inefficient healthcare provider access to health information, which contributes to a cascade of poor information flow to residents and their families, particularly around medications [1, 3].

For residential aged care clients specifically, the information needs we identified in this study can largely be met by the implementation of a well-designed dashboard which could present relevant information in (near) real-time. As aged care facilities increasingly adopt digital clinical systems, medication information would be readily available and the development of a dynamic dashboard that collates this information and presents it to users in a simple way, would be feasible [41]. Having a dynamic dashboard that is accessible at any time, such as through an online portal, would enable residents and their families to access trusted information readily when they need it. This is particularly important as it was stated by participants that decision-making in aged care is shared amongst the resident, family members and healthcare providers of the resident [3, 4].

Other than participants who expressed little interest in a dashboard, most saw value in the digital delivery of health information, supporting Siette et al.’s finding that older adults report a medium-high acceptability of digital dashboards [21]. This is particularly interesting given that comfort levels regarding using technology varied amongst the participants of this study. Further, there has been an increase in older adults’ technology use in recent years, particularly since the start of the COVID-19 pandemic which shifted the way older adults received information and connected with society [42]. In Australia, 93% of older adults (aged 64 years or older) now having access to home Internet [43]. Moreover, the Australian Communications and Media Authority reported that in 2022, 54% of Australians aged 65–74 years, and 47% of Australians aged 75 year or older, accessed the Internet at least weekly via a laptop, with 88% of 64–75-year-olds and 70% of > 75-year-olds accessing the Internet via mobile phones [44]. Given this study was conducted before COVID-19, in the post COVID-19 context, older adults who may have been less familiar with technology are now more adept at navigating such systems with Sixsmith and colleagues’ finding that > 68% of older adults indicated they will continue to use technologies they adopted during lockdowns in a post-pandemic era [45]. This suggests that aged care client and consumer preferences are increasingly relevant considering the rapidly evolving digitalisation of the aged care industry during and after the COVID-19 pandemic. Some participants in this study did propose having a paper-based option of the dashboard, i.e., print-outs. For residential aged care, participants recommended family member involvement in viewing the dashboard, as well as providing dashboard training for clients. Moyle and colleagues’ qualitative survey of Australian older adults, family members, healthcare professionals and aged care providers, identified the need for enhanced training to support aged care clients’ digital literacy and use of digital technologies [46]. They recommended that this could be achieved through user-friendly guides, practical sessions and vision tutorials.

There are several advantages to having a digital dashboard in aged care. A digital dashboard can be customised for multiple end-users’ preferences [3, 41, 47, 48]. Participants agreed that the dashboard should be easy to use and understand, i.e., simple, jargon-free and easy-to-read text. This finding echoes Moyle et al.’s study where older adults, family members and providers advocated for “simpler” technology for aged care consumers [46]. Siette’s review of usability and acceptability of clinical dashboards in aged care found that older adults tended to report usability of dashboards as low [21]. Moyle et al. recommended that digital technologies should be co-designed with key stakeholders in aged care to produce user-friendly technologies [46]. Participants in the current study were also supportive of the use of colours in the dashboard, when used appropriately, for example, use of the familiar traffic-light system. The red/yellow/green is a “universally understood” system for communicating risk and a common feature in clinical dashboards [7]. Aged care clients did have different preferences regarding how they wanted information presented to them, such as the amount of data that participants requested be displayed, which is typical for information quantity requirements of dashboard in general [49]. Several participants in this study stated that they wanted more information, while others wanted more succinct data. A dashboard could alleviate this issue through the use of expandable information boxes or ‘drill-through’ functionalities [24, 48, 49]. These boxes would work in a way where only summarised information is initially presented to end-users and then if end-users wanted further information, they could click on a button that expanded the box and provided them with further information related to the summary. Similarly, inclusion of graphs, could be “hidden” until an end-user who wishes to see a graphical depiction of information opens it [24, 48, 49]. These are just some examples, of how a dynamic dashboard could benefit multiple end-users in accessing health information, consistent with guidelines for optimal dashboard development [22, 24].

### Strengths and limitations

A strength of this study was that it details one of the initial phases of a user-centred design approach in informing a consumer-facing aged care dashboard. User-centred design is important as the process involves end-users in the development of products that are intended to be used by them, commonly used in dashboard development [47, 48, 50]. Therefore, understanding end-users’ needs and preferences and then tailoring products accordingly is critical to ensure increased uptake of health technologies. This study sought feedback from various aged care service users (both community-based and those living in residential aged care) and their family members, who often play an important role in decision-making about care. However, this study also had limitations, including a small sample of aged care residents and family members, the latter of whom are likely to be primary dashboard users. It was originally intended for interviews to be conducted face-to-face. Due to the COVID-19 pandemic, interviews had to be held over the telephone, which limited the participant pool to those who were able to use a telephone independently. This may have affected the number of residential aged care residents and subsequent family members we were able to recruit to reach data saturation, and thus the generalisability of results to other aged care residents and their informal caregivers.

### Implications and future directions

This study provides the foundation for an ongoing and iterative co-design process with community and residential aged care clients and their family members to develop, implement and evaluate an aged care digital dashboard, known to be effective in dashboard design [50]. This study demonstrated that whilst interest in such a tool is limited, aged care clients and their families have positive views of a planned digital dashboard for their use in aged care. While participants generally shared similar views of content, appearance and delivery of the dashboard, differences in preferences were found. Individual differences included preferences regarding the use of graphs, colours and symbols, for example. The next steps in this research project include designing prototypes of the dashboard based on the suggestions generated from this study and then presenting these prototypes to aged care clients, residents, and family members for their feedback. This would then trigger another round of iterations to the dashboard, before pilot implementation in an aged care setting using a cluster-randomised controlled trial to investigate its usability and acceptability for end-users.

## Conclusions

Aged care clients and their family members hold generally positive views about a clinical dashboard that presents timely and accurate information to them and is accessible when they require it. With customisation to user group preferences, such a dashboard would support their current practices of information-seeking which included requesting information from relevant healthcare providers and self-seeking, such as searching the Internet. In terms of design of a dashboard, aged care clients and their family members provided a plethora of suggestions for a digital dashboard that need to be further synthesised before being used by various aged care service users. Many of these recommendations, such as inclusion of graphs, use of colour and access to additional informational websites, will be incorporated into the subsequent design phases of an aged care digital dashboard.

## Electronic supplementary material

Below is the link to the electronic supplementary material.

Supplementary Material 1

## Data Availability

The datasets used and/or analysed during the current study available from the corresponding author on reasonable request.

## Abbreviations

GP: General practitioner
PRN: Pro re nata
