# Supplementary material for: Using co-design to understand consumer’s health information-seeking behaviours and design preferences for a new digital clinical dashboard in aged care
Source: BMC Geriatr. 2024 Dec 4;24:993. doi: 10.1186/s12877-024-05581-2 (PMC11616187; doi:10.1186/s12877-024-05581-2)
Supplement: Supplementary file 1 — Supplementary Material 1 [file 12877_2024_5581_MOESM1_ESM.docx]

# Community care clients – Baseline interview guide

## Client/Carer information seeking and dissemination

Our research team is trying to understand what types of health and care information you use, and how to best use this information to support delivery of care to you/your family member.

1. Please describe some situations when you would look for health and care information for you/your family member’s care.
2. How much health and care information do you like to have in general? Why?
3. How involved do you like to be in your own/family member’s clinical and care decisions?
   1. What do you think are the most important aspects of care you should have a say in?
4. When you make clinical and care decisions, such as asking a doctor about medicines you/your family member is on, what information:
   1. Do you currently use?
   2. Do you currently not have, but wish you did?
   3. Of all the information you mentioned, which is the most important to you to be able to make clinical decisions? Why?
5. How do you access health and care information?
   1. What information is currently:
      1. Hard to access? Why is it hard?
      2. Easy to access? Why is it easy?
      3. Hard to understand? Why is it hard?
      4. Easy to understand? Why is it easy?
6. How comfortable are you with using technology?
   1. Can you tell me more about why you are comfortable/uncomfortable?
   2. What devices do you use?
   3. In a day, how often do you use these devices?

## Dashboard preferences

Our research team is developing a dashboard to help support the management of aged care clients. A dashboard is an ‘at a glance’ summary of health information, that can be used by people such as doctors and nurses, and clients and their families. The dashboard will help to identify clients who are at risk of poor health such as an increased chance of falling or low wellbeing. We want to make the dashboard as useful as possible for clients such as yourself and your family members.

1. The dashboard will display your/your family member’s care and health information This may include their medications, participation in social events, meals and health conditions. If you used an ‘at a glance’ summary of your/your family member’s care and health information, what would you like the dashboard to tell you?
2. Would you like the dashboard to be electronic or paper-based? Or would you like it presented in another way? Why?
   1. If electronic: How do you want to access the electronic dashboard?
   2. If paper: How would you like to receive this?
      1. How often would you want to receive the dashboard summary?
   3. If other: Why do you prefer it presented this way?
3. Now I will ask you a few questions about how you want the dashboard to look. Please explain how you feel about:
   1. The use of colours to show areas you/your family member are doing well or not well in?
   2. The amount of text that should be used?
   3. The size of text that should be used?
   4. The use of pictures or symbols?
   5. The use of graphs, for example to see changes over time?
   6. The amount of information the dashboard should show?
   7. The option to contact a nurse to discuss the dashboard?
4. Do you have any other comments or suggestions about this research?
